# Machine-Learning Guided Discovery of Bioactive Inhibitors of PD1-PDL1 Interaction

**DOI:** 10.3390/ph15050613

**Published:** 2022-05-16

**Authors:** Sachin P. Patil, Elena Fattakhova, Jeremy Hofer, Michael Oravic, Autumn Bender, Jason Brearey, Daniel Parker, Madison Radnoff, Zackary Smith

**Affiliations:** 1 NanoBio Lab, School of Engineering, Widener University, Chester, PA 19013, USA; 2 Department of Chemical Engineering, Widener University, Chester, PA 19013, USA; efattakhova@widener.edu (E.F.); arbender@widener.edu (A.B.); jjbrearey@widener.edu (J.B.); dbparker@widener.edu (D.P.); maradnoff@widener.edu (M.R.); zrsmith@widener.edu (Z.S.); 3 Department of Computer Science, Widener University, Chester, PA 19013, USA; jhofer@widener.edu; 4 Department of Biomedical Engineering, Widener University, Chester, PA 19013, USA; moravic@widener.edu

**Keywords:** cancer immunotherapy, protein-protein interaction, immune checkpoint, PD1-PDL1, PDL1 dimerization, machine learning, random forest, 2D fingerprints, molecular dynamics

## Abstract

The selective activation of the innate immune system through blockade of immune checkpoint PD1-PDL1 interaction has proven effective against a variety of cancers. In contrast to six antibody therapies approved and several under clinical investigation, the development of small-molecule PD1-PDL1 inhibitors is still in its infancy with no such drugs approved yet. Nevertheless, a promising series of small molecules inducing PDL1 dimerization has revealed important spatio-chemical features required for effective PD1-PDL1 inhibition through PDL1 sequestration. In the present study, we utilized these features for developing machine-learning (ML) classifiers by fitting Random Forest models to six 2D fingerprint descriptors. A focused database of ~16 K bioactive molecules, including approved and experimental drugs, was screened using these ML models, leading to classification of 361 molecules as potentially active. These ML hits were subjected to molecular docking studies to further shortlist them based on their binding interactions within the PDL1 dimer pocket. The top 20 molecules with favorable interactions were experimentally tested using HTRF human PD1-PDL1 binding assays, leading to the identification of two active molecules, CRT5 and P053, with the IC_50_ values of 22.35 and 33.65 µM, respectively. Owing to their bioactive nature, our newly discovered molecules may prove suitable for further medicinal chemistry optimization, leading to more potent and selective PD1-PDL1 inhibitors. Finally, our ML models and the integrated screening protocol may prove useful for screening larger libraries for novel PD1-PDL1 inhibitors.

## 1. Introduction

The majority of the important biological functions in living organisms including humans are regulated and executed through a vast array of protein–protein interactions (PPIs). In this context, the human interactome may represent a promising source of many novel therapeutic targets owing to the large size of the human interactome estimated to range from ~300,000 [1] to as high as ~650,000 PPIs [2]. To date, the interactome engineering through PPI modulation has been under the realm of primarily large biologics-based therapies, including antibodies and fusion proteins. This is due to the relatively large, featureless protein–protein binding interfaces lacking the well-defined “druggable” binding pockets that are the hallmarks of the traditional targets like G-protein coupled receptors (GPCRs) and enzymes [3]. These large biotherapeutics, however, possess several inherent drawbacks such as lack of oral bioavailability, low tumor infiltration, adverse immune events, and high cost. These drawbacks can be successfully overcome with the use of low molecular weight (up to 500 Da) bioactive chemical compounds, and small-molecule tractability of PPIs is on the rise with the first such bioactive drugs entering clinical trials recently [4].

One such important PPI that has been clinically targeted by humanized monoclonal antibodies and warrants development of viable small-molecule alternatives is the programmed cell death protein 1 (PD1)–programmed cell death ligand 1 (PDL1) interaction. The PD1 is an immunoinhibitory receptor expressed on the surface of activated T and B cells, while its ligand PDL1 is present on different cell types, including activated T and B cells, dendritic cells, macrophages, and mesenchymal cells [5]. Notably, many tumor types, such as melanoma and carcinomas of bladder, breast, colorectum, head, kidney, liver, lung, neck, ovary and pancreas, also overexpress PDL1 on their cell surfaces [6]. Thus, although PD1-PDL1 interaction is physiologically beneficial in preventing excessive stimulation of immune system, it also leads to detrimental immune tolerance within the tumor microenvironment through T-cell functional exhaustion and apoptosis, in turn helping the tumors escape their immune destruction. Therefore, pharmacological inhibition of PD1-PDL1 inhibition has emerged as one of the most promising therapeutic strategies to strengthen the immune response against a broad-spectrum of PDL1-expressing cancers [7].

To date, the U.S. Food and Drug Administration (FDA) has approved seven monoclonal antibodies exhibiting unprecedented clinical benefits through effective PD1-PDL1 blockade by binding to either PD1 (Cemiplimab, dostarlimab, Nivolumab, and Pembrolizumab) or PDL1 receptors (Atezolizumab, Avelumab, and Durvalumab) [8]. In contrast, development of small-molecule PD1-PDL1 inhibitors has lagged considerably, owing to the inherent lack of druggability of such traditionally challenging PPIs with large yet featureless binding interfaces. The PD1-PDL1 interface is especially challenging to target using small molecules because of a large interface spanning over ~1970 Å^2^ that is devoid of well-defined binding pockets [9]. Accordingly, efforts to directly target this PD1-PDL1 binding interface have yielded only modestly active small-molecule inhibitors with µM activities [10,11]. Another novel approach utilized by Bristol Myers Squibb (BMS) to block PD1-PDL1 interaction involves PDL1 dimerization, yielding small molecules with impressive IC_50_ values in the pM-nM range [12]. These novel molecules effectively block the PD1-binding pockets on PDL1 proteins by forming and stabilizing the PDL1 homodimers [13]. The clinical development of these potent PDL1 dimerizers, however, is still in its infancy, with only one such molecule (INCB86550 from Incyte, Wilmington, DE, United States) reaching early clinical trials (NCT03762447, clinicaltrials.gov, accessed on 10 April 2022, Bethesda, MD, USA), and none being approved yet.

Thus, there is a significant unmet need to explore this highly promising but relatively uncharted therapeutic space for potential cancer immunotherapy applications. In the present study, we aimed to discover bioactive PDL1 dimerizers for PD1-PDL1 blockade via machine-learning (ML) enabled virtual screening combined with in vitro experimental testing.

## 2. Results and Discussion

**Figure 1:**
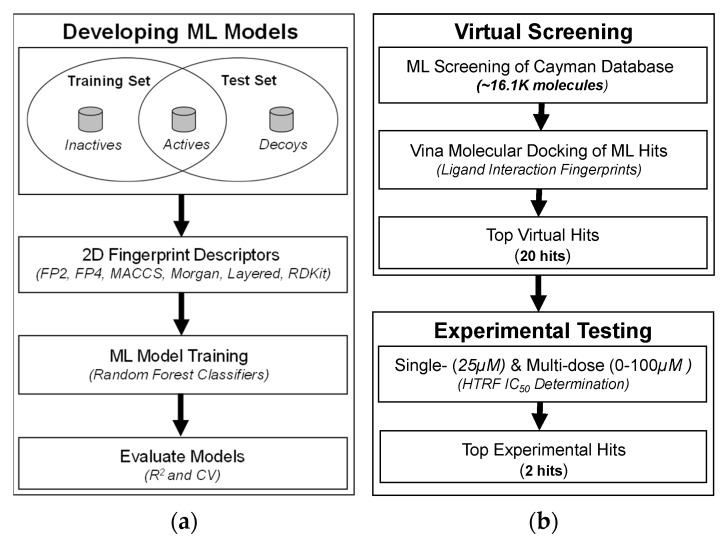
Overview of the integrated screening protocol to identify small-molecule inhibitors of PD1-PDL1 interaction: (**a**) Development of ML models based on 2D fingerprint descriptors of known ACTIVE and INACTIVE molecules against PDL1; (**b**) ML-based virtual screening, followed by experimental testing of top virtual hits using HTRF assay.

The process of new drug discovery, design and development remains immensely challenging due to inherent high cost and time requirements. The success rate of this challenging process can be amplified when a combination of computational and experimental approaches is utilized. Recently, computer-aided drug discovery process has been improved by utilizing various machine-learning approaches [14]. Here, we developed ML models based on 2D chemical descriptors to optimize virtual screening followed by experimental testing for bioactive PD1-PDL1 inhibitors. The schematic of our integrated virtual and experimental screening protocol is shown in Figure 1.

We started by analyzing the promising series of novel small-molecule PDL1 dimerizers invented by the Bristol-Myers Squibb (BMS) and published in 6 patents [WO2015034820A1; WO2015160641A2; WO2018009505A1; WO2018183171A1; WO2017066227A1; WO2018044963A1]. These potent PDL1 dimerizing molecules have revealed important spatio-chemical features required for PDL1 sequestration, leading to effective PD1-PDL1 inhibition. In the present study, we utilized these molecular features for developing ML classifiers by fitting Random Forest (RF) models to 2D fingerprints descriptors of these known PDL1 ligands. Specifically, six fingerprint descriptors (FP1, FP2, Layered, MACCS, Morgan, RDKit) implemented in the Open Drug Discovery Toolkit (ODDT) [15] were generated for 1581 BMS molecules (classified as “ACTIVE”), their property-matched DECOYS (50 DECOYS per ACTIVE molecule) obtained from DUD-E database [16], and 417 known INACTIVE molecules from our in-house experimental studies against this target (Supplementary Table S1). The objective was to utilize these multiple fingerprint descriptors for describing active compounds for the PDL1 dimerization approach in comparison to the compounds that we have previously found to be inactive in inhibiting the PD1-PDL1 interaction.

In this context, we first applied ML RF classifiers on 2D molecular fingerprint descriptors of these ACTIVE and INACTIVE molecules (Figure 1). The quality of these trained models was assessed by a correlation coefficient (R) implemented in the ODDT, with Morgan fingerprints yielding the best results against both the training (R^2^ = 0.9729) and the test (R^2^ = 0.9664) sets, while other fingerprints also following closely (Table 1).

Therefore, we decided to use all of these six fingerprint models to carry out ensemble screening of a commercial database for identifying potential PDL1 dimerizers for PD1-PDL1 inhibition. The Cayman Chemical database contained 16,191 bioactive molecules containing many approved and experimental drugs. The ensemble screening of Cayman database using our machine-learning models led to classification of 361 molecules as potentially active against PD1-PDL1 target by at least one out of six fingerprint models. This computational screening output with ~98% reduction in database size is helpful in potentially limiting the number of inactive and false positive molecules that would be tested in the wet-lab experiments.

**Figure 2:**
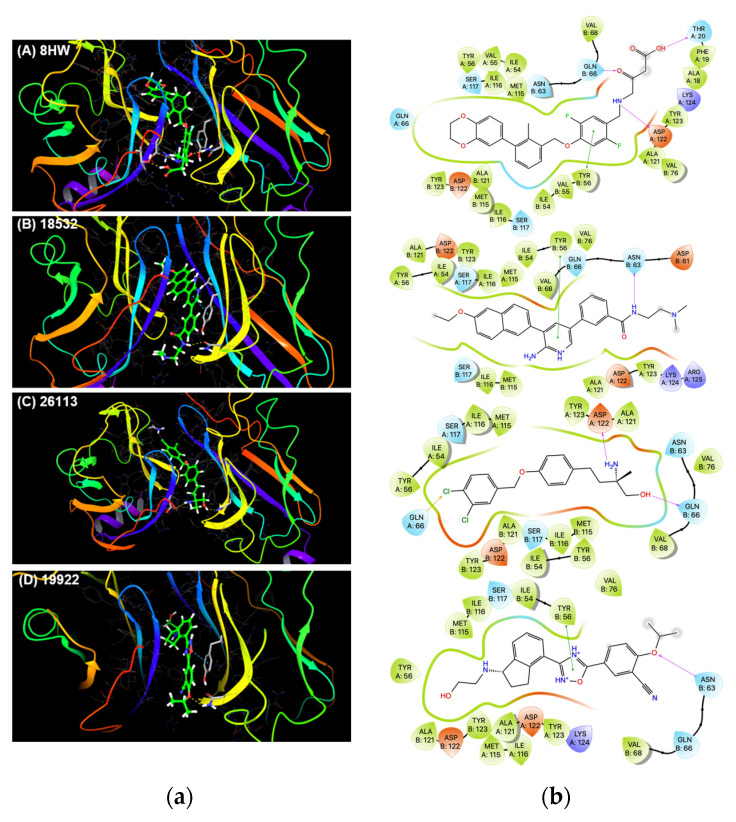
The PDL1 dimer binding modes of crystal ligand (8HW) and three hits (18532, 26113, and 19922) predicted by AutoDock Vina: (**a**) Representation of PDL1 dimer pockets containing bound ligands; and (**b**) 2D ligand–protein interactions.

To further reduce the number of molecules for experimental testing, we subjected these 361 ML hits to structure-based docking studies against PDL1 dimer pocket. Many X-ray crystal structures of PDL1 dimers bound with their respective crystal ligands are available with varying resolutions ranging from 1.70 Å to 2.79 Å (PDB IDs: 5N2F, 5NIU, 6R3K, 5J89, 5J8O, 5N2D, 6NM8), with 5N2F having the highest crystallographic resolution (1.70 Å). Previously, we have carried out ensemble virtual screening against all 7 receptors, leading to identification of Pyrvinium, an FDA-approved anthelmintic drug, as a small-molecule PD1-PDL1 inhibitor with IC_50_ value of ~29.66 μM [17]. Among all 7 published crystal structures, 5N2F was found to exhibit the highest average docking score for all 7 PDL1 crystal ligands, thus proving it suitable for our current molecular docking studies. Therefore, in the present study, we docked our 361 ML hits against only this highest resolution structure 5N2F. The AutoDock Vina algorithm [18] was used to carry out docking, which correctly predicted binding mode of the crystal ligand 8HW with −11.4 Kcal/mol binding score against 5N2F (Figure 2).

The structural interaction fingerprints [19] for top-ranking poses for all the docked compounds and known PDL1 crystal ligands were then generated using the script implemented in Schrödinger’s graphical user interface Maestro. The PDL1 crystal ligands showed various key interactions potentially responsible for their potent receptor-binding ability and corresponding IC_50_ values. These included aromatic π-π interactions with Tyr56 and Tyr123 on both PDL1 chains of the dimer, hydrophobic interactions with several amino acids along the tunnel-like pocket of PDL1 dimer, and hydrogen and halogen bonds at the solvent-exposed opening of the pocket. The hydrogen bond interactions with Asp122, Lys124 and Arg125 have shown to play important role in ligand binding to PDL1 [20]. Therefore, we selected ligands exhibiting similar interactions with the PDL1 pocket. In addition, we visually inspected top-ranking poses of all the docked ligands for their proper docking modes within the PDL1 pocket and their alignment in comparison to the crystal ligand 8HW. Through these structure-based analyses, we shortlisted a total of 20 molecules to investigate their potential activity in inhibiting PD1-PDL1 interaction in vitro (Table 2).

The homogeneous time-resolved fluorescence (HTRF) binding assay was used to test these top 20 molecules for their potential blockade of human PD1-PDL1 interaction. We have previously established the utility of the HTRF assay over similar homogenous assay like AlphaLISA, with the latter proving to be more promiscuous in identifying potential false positives against PD1-PDL1 target [17]. Importantly, a known PD1-PDL1 inhibitor (BMS-1166) was used as a positive control to investigate the compatibility of this assay for the present experimental screening. BMS-1166 induced dose-dependent inhibition of PD1-PDL1 interaction with the IC_50_ value of ~1.0 nM that is in agreement with its reported HTRF IC_50_ range of 0.06–10 nM [21]. This further supported the use of the HTRF assay for our current studies, leading to identification of 3 out of 20 compounds exhibiting ≥30% PD1-PDL1 inhibition at 25 µM single-dose test concentration (Table 2).

**Figure 3:**
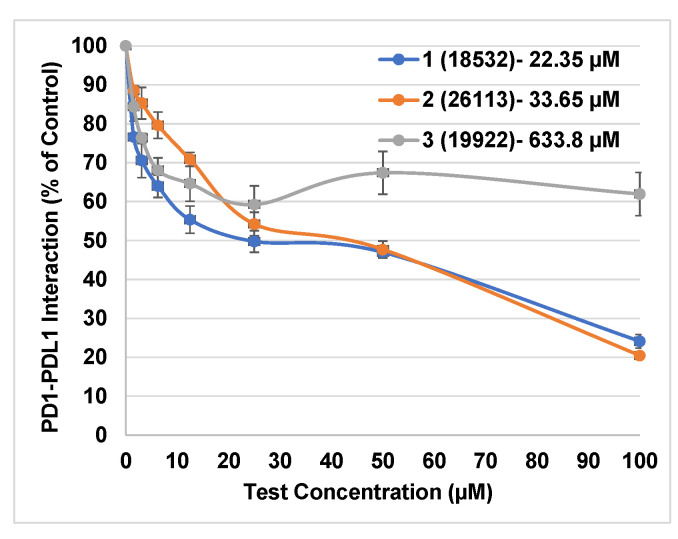
Dose–response HTRF data for small-molecule inhibitors of PD1-PDL1 interaction.

We subjected these 3 active compounds to dose-dependent experimental testing to further confirm their observed activity. Two out of these 3 compounds showed promising dose-dependent activity, with the HTRF IC_50_ value of the most active compound **1** being ~22 µM (Figure 3). This is a promising activity by a relatively low-molecular-weight compound **1** (454.6 g/mol) against a large protein–protein interaction like PD1-PDL1 with a large binding interface spanning ~1970 Å^2^ [22]. It is also noteworthy that both compounds **1** and **2** have been shown to be active in cell-based and animal model studies of different human indications, thus emphasizing their bioactive nature against important therapeutic targets. Specifically, compound **1** (CRT5) is a specific protein kinase D (PKD) inhibitor with 1–2 nM IC_50_ values against all three PKD isoforms in VEGF-treated endothelial cells [23]. Compound **2** (P053) is a potent, selective ceramide synthase 1 (CerS1) inhibitor leading to increased fatty acid oxidation in skeletal muscles of mice fed with high-fat diet [24]. Our present study is the first to show the potential role of these bioactive compounds as small-molecule PD1-PDL1 inhibitors, warranting their further investigation for potential anti-cancer benefits.

**Figure 4:**
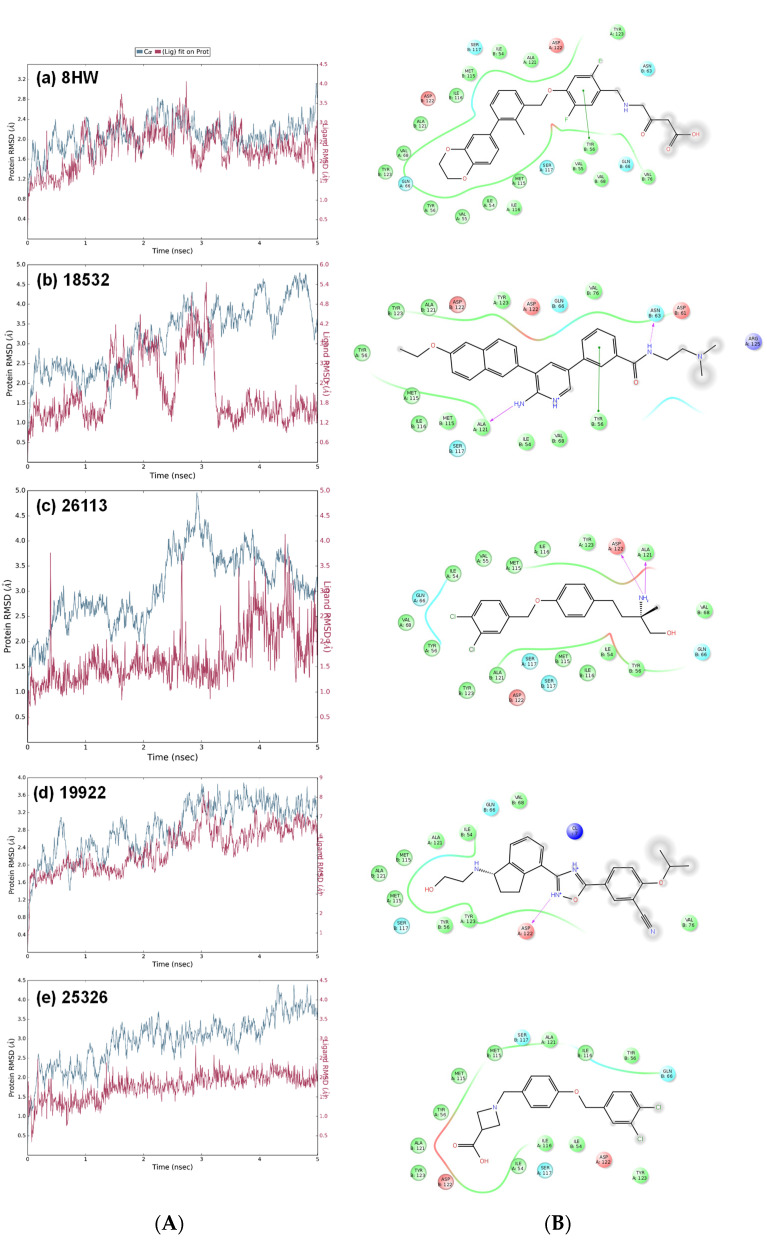
The MD simulation data for crystal ligand (8HW), three hits (18532, 26113, and 19922), and a structural analog (25236): (**A**) The RMSD analyses over 5000 ps molecular dynamics simulation of ligand binding in the PDL1 dimer pocket; and (**B**) the ligand–protein interaction diagram at the end of MD simulation.

To study possible molecular interactions and binding stability of our active compounds in comparison to the crystal ligand 8HW within the PDL1 dimer interface, their top-ranked Vina docking complexes were subjected to molecular dynamics simulations (Desmond Molecular Dynamics System from D. E. Shaw Research, New York, NY, USA). The root mean square deviation (RMSD) analysis of the best active compounds **1** and **2** over 5 ns molecular dynamics simulation revealed their stable binding within the PDL1 dimer pocket (Figure 4A). Our top hit compound **1** (IC_50_~22.35 µM) showed similar binding stability as the crystal ligand 8HW. After initial configuration change, compound **1** did not deviate much, exhibiting consistent RMSD value around ~2.0 Å at the end of the 5 ns simulation period, thus indicating its stable binding at the PDL1 dimer interface. The ligand RMSD value for compound **2** was ~3.0–4.0 Å, which is consistent with its slightly higher IC_50_ value of ~33.65 µM. Here, it should be noted that the RMSD profile for ligand-bound PDL1 backbone for both compounds **1** and **2** fluctuated as high as ~5.0 Å, as compared to observed ligand–protein stability in case of crystal ligand 8HW. For compound **3** (19922), a high ligand RMSD value of ~8.0 Å was observed, coinciding with its very high IC_50_ value of ~633.80 µM.

The analysis of the final pose of the ligand-complex after 5 ns molecular dynamics simulation revealed several key interactions between our best active compound **1** with the PDL1 dimer pocket (Figure 4B). Specifically, compound **1** is predicted to make hydrophobic interactions with several amino acid residues lining the PDL1 dimer tunnel. Importantly, naphthalenyl moiety in compound **1** occupied the distal end of the PDL1 dimer pocket, functionally replacing the biaryl moiety present in the published PDL1 dimerizers including the crystal ligand 8HW (Figure 4B). Furthermore, the amine group in the middle pyridinyl moiety formed H-bond with Ala121 amino acid residue. The π-π interaction with Tyr56 amino acid residue is also observed in case of compound **1**, which is a well-known characteristic of published PDL1 dimerizers, including crystal ligand 8HW (Figure 4B).

Notably, the dimethylamino-ethyl-benzamide tail part of compound **1** is predicted to make a hydrogen bond with the PDL1 pocket residue exposed to the solvent (Asn63), further contributing to the ligand–PDL1 binding. The importance of such tail part H-bond interactions for the observed activity is evident by comparing structure of the active compound **2** with a structurally similar compound **19** (25326) that completely lacked activity against PD1-PDL1 interaction (Table 2). The difference between these two structural analogs is the lack of such tail-part H-bond capabilities in the inactive compound **19** versus the active compound **2** (Figure 4B). This small structural difference completely changed the binding mode of compound **19** with its orientation shifting in 180°, with dichlorobenzyl group exposed to solvent, as opposed to filling the hydrophobic PDL1 dimer pocket.

**Figure 5:**
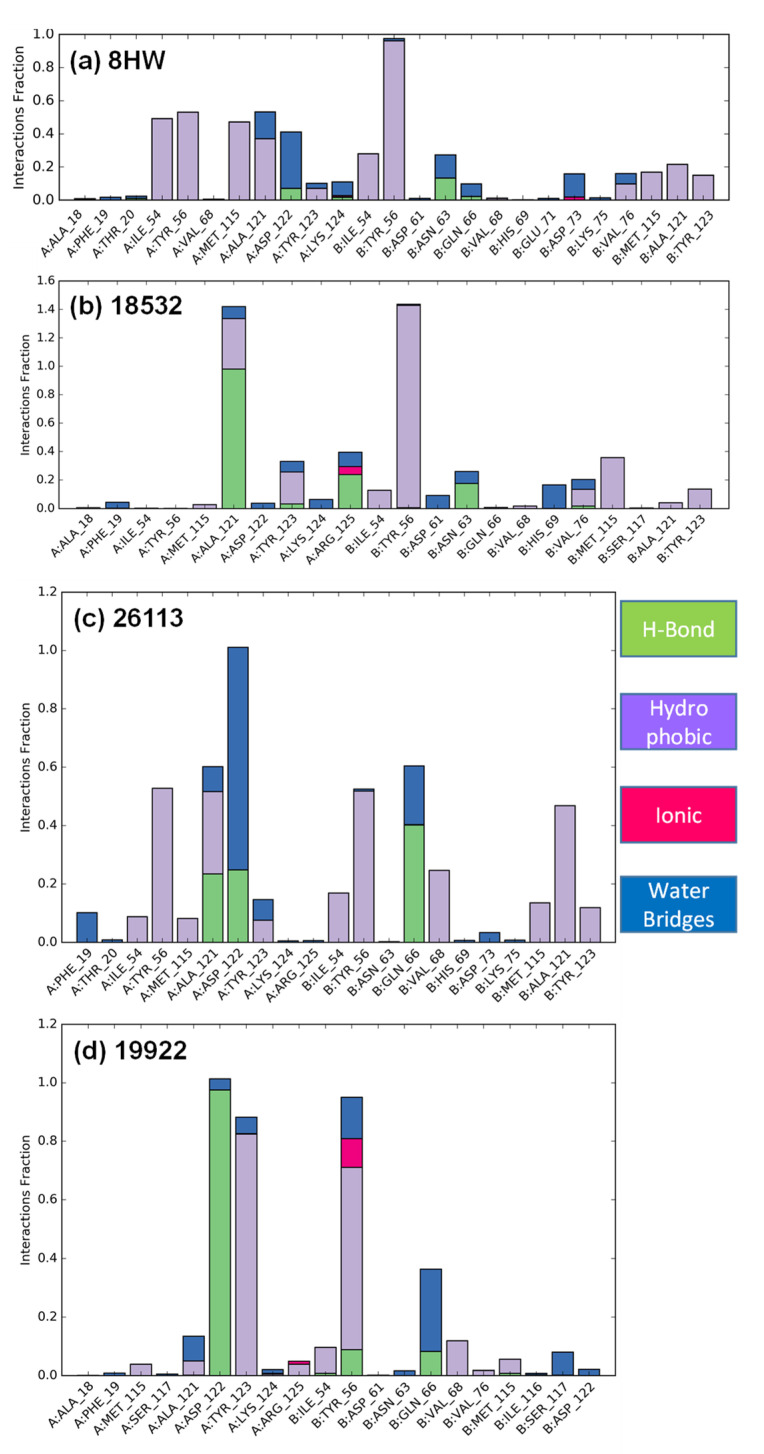
The protein interactions with the ligand (H-bonds, hydrophobic, ionic and water bridges) normalized over the course of the simulation. Our most active compound **1** (18532) exhibited strong interactions with two key amino acids (Tyr56 and Ala121).

In contrast to active compounds **1** and **2**, compound **3** did not bind well with the PDL1 dimer, resulting in an unstable ligand–protein complex over 5 ns simulation period (Figure 4A). This is in line with the lack of key aromatic, hydrophobic, and H-bond interactions between compound **3** and the PDL1 dimer residues (Figure 4B). Notably, as compared to crystal ligand and our two top active compounds **1** and **2**, compound **3** showed minimal hydrophobic interactions and complete lack of H-bond interactions with the Ala121 residue (Figure 5). These molecular modeling data further support the experimentally observed weak PD1-PDL1 inhibitory activity of compound **3** with IC_50_ value of ~633.80 µM (Figure 3).

In summary, our present study involving ML-enabled virtual screening and in vitro experimental testing successfully identified compounds **1** (CRT5) and **2** (P053) as potential inhibitors of the immune checkpoint PD1-PDL1 interaction.

## 3. Materials and Methods

### 3.1. Development of 2D Fingerprint-Based Classification Models

A key goal of our experimental design was to train a classifier machine learning (ML) models that could preprocess a commercial molecular database for potential active compounds against PD1-PDL1 interaction. To achieve this, six BMS patents published in the literature were mined to obtain the SMILES for the small molecule PDL1 dimerizers (classified as “ACTIVE”). First, the IUPAC (International Union of Pure and Applied Chemistry) names of ACTIVE molecules were text mined from BMS patents and then corresponding SMILES (simplified molecular input line entry specification) were obtained using OPSIN (Open Parser for Systematic IUPAC Nomenclature) [25]. A total of 1581 ACTIVE molecules were thus compiled with varying HTRF IC_50_ values, ranging from 0.6 nM to 100 µM. With the aim of developing classification models, inclusion of another group of molecules is warranted in addition to these ACTIVE molecules. Therefore, we included 417 molecules that were unable to inhibit PD1-PDL1 interaction in our previous in-house experimental work (classified as “INACTIVE”). Six fingerprint descriptors (FP2, FP4, MACCS, RDKit, Morgan, Layered) implemented in the ODDT [15] were generated for these actives, their property matched DECOYS from DUD-E database [16], and experimentally established INACTIVE molecules against PD1-PDL1 target. The ML Random Forest (RF) classifier model was then fit on the molecular fingerprints in the training (ACTIVE and INACTIVE) and test (ACTIVE and DECOYS) sets. The RF classification is an ensemble based classification method, which involves generation of a number of internal models with introduced randomness to create naturally varying results. Each of these RF classifier models is executed on the provided data to generate a classification for the supplied input. The results from all the internal models thus trained are then averaged, resulting in a final RF model result. The models internally used here are Decision Trees, which are supervised learning methods whose goal is to generate a resulting classification based on a number of dynamically generated decisions based on the data utilized to train the decision tree. Here, we utilized the ODDT platform to facilitate creating the RF classifiers through use of the open source Python library Scikit-learn. For each type of fingerprint generated, a different RF classifier was trained, being provided the fingerprints of each input molecule for the given fingerprint type as well as the corresponding classification (“ACTIVE”, “INACTIVE”) of each molecule. Parameters supplied for the RF classifier training included: n_estimators = 500, oob_score = true, max_features = 6, min_samples_split = 6. Default values were used for all other parameters. The quality of the ML RF models thus trained on our data was assessed by a correlation coefficient (R) implemented in the ODDT. Specifically, cross validation algorithms implemented in ODDT via Scikit-learn were performed on the generated RF models to evaluate their performance. The models with >90% accuracy in cross validation were considered a good fit. Finally, the trained RF classifier models based on six fingerprint models were utilized to screen the fingerprint models generated for 16,191 bioactive molecules from Cayman Chemical database. The RF classifiers returned a generalized “ACTIVE” or “INACTIVE” classification for these molecules. Top 361 molecules thus classified as “ACTIVE” by at least 1 out of 6 fingerprint models were then subjected to further computational and experimental studies.

### 3.2. Molecular Docking and Ligand Interaction Fingerprint Analysis

The SMILES for top 361 ML hits were used to generate their 3D conformers using OMEGA 4.1.2.0: OpenEye Scientific Software, Santa Fe, NM (http://www.eyesopen.com, accessed on 10 April 2022) [26]. The docking of these molecules was then carried out against a published crystal structure of the PDL1 dimer with the best resolution of 1.70 Å (PDB ID: 5N2F), downloaded from the protein data bank (PDB) [27] and processed using AutoDock Tools [28]. The AutoDock Vina docking algorithm [18] was used to carry out the structure-based docking of these bioactive molecules into the PDL1 dimer interface. The docking search space coordinates used for docking were: [Center: X: 31.9429, Y: 12.7403, Z: 133.7878; Dimensions (Å): X: 20.0, Y: 20.0, Z: 20.0]. Default AutoDock Vina docking parameters were used, and the ligands were ranked according to their best docking scores. The protein–ligand binding interactions of all the docked compounds were analyzed using the structural interaction fingerprint (SIFt) method [19]. The SIFt analysis was used to shortlist compounds that make aromatic π-π interactions and H-bond interactions with key amino acids including Tyr56, Asp122, Lys124 and Arg125, which are shown to be important for PDL1 ligand binding [20]. These top binding poses of these shortlisted compounds were further inspected visually for their proper binding orientation within the PDL1 dimer pocket, leading to their selection for the experimental testing.

### 3.3. Molecular Dynamics Simulations

To investigate ligand binding stability, molecular dynamics (MD) simulations were conducted on respective ligand–protein complexes using Desmond Molecular Dynamics package (D. E. Shaw Research, New York, NY, USA). The top binding conformation from AutoDock Vina docking was used as the starting conformation for MD simulation. The ligand interactions were modeled with the OPLS_2005 force field. The TIP3P water model was used to solvate the ligand–protein complex, setting up the system for MD simulations.

The solvated system was further neutralized using an appropriate number of counterions. Default parameters in Desmond were utilized to equilibrate this molecular system. Finally, the equilibrated system was subjected to 5 ns MD simulations at constant temperature and pressure of 300 K and 1 atm, respectively. The atom coordinates were recorded at every 5.0 ps for the follow-up MD simulation analyses. The root mean square deviations (RMSDs) for the protein and the docked ligand were calculated over the entire simulation trajectory with reference to their respective first frame. The protein interactions with the ligand were normalized over the course of the 5 ns trajectory, with the score value depicted in the stacked bar chart indicating the percentage of the simulation time when a specific interaction is maintained. These score values may exceed 1.0 (or 100%), as a given protein residue may make multiple contacts of the same subtype with the ligand.

### 3.4. In Vitro Testing Using HTRF Assay

The potential activity of our virtual hits in inhibiting PD1-PDL1 interaction was investigated using the homogeneous time-resolved fluorescence (HTRF) binding assay from Perkin Elmer. The assay was carried out according to the manufacturer’s instructions using the HTRF protocol in BioTek Synergy Neo^TM^ microplate reader. The percent inhibition of PD1-PDL1 interaction by test compounds was calculated by subtracting the assay signals for the compounds at 25 μM concentration from the control (untreated) signal. The assay mixture with only PDL1 protein, but not PD1 protein served as a negative control denoting 0% PD1-PDL1 interaction. Dose–response curves (0–100 µM) were generated for top three active compounds exhibiting >30% inhibition at 25 μM test concentration. The dose–response data were analyzed using GraphPad Prism to determine IC_50_ values using nonlinear regression variable slope models.

## 4. Conclusions

In the present study, a ML-based virtual screening approach was applied to screen a commercially available database for potentially novel inhibitors of PD1-PDL1 interaction. Several molecules were identified with 2D chemical fingerprints similar to the known PDL1 dimerizers and hence classified as possibly “Active” for PD1-PDL1 blockade. The RF ML predictions were further shortlisted to the top 20 virtual hits using ligand–receptor interaction fingerprint analysis. The experimental testing of these computational hits using in vitro HTRF assay led to identification of two structurally novel molecules with PD1-PDL1 inhibition activity in the µM range. Despite having relatively weaker µM activities than the known PD1-PDL1 inhibitors with nM activities, the newly identified experimental compounds have the advantage of being tested in cell and animal models of human clinical importance [23,24]. It is noteworthy here that the known PD1-PDL1 inhibitors, although potent, are associated with acute cytotoxicity [29], thus hampering their pre-clinical and clinical development. Thus, there is an unmet need to identify and design alternative PD1-PDL1 inhibitors, including PDL1 dimerizers, with the aim of overcoming this developmental hurdle. In this context, our two bioactive hits may prove suitable as starting points for further hit-to-lead optimization in the quest for design and development of more potent and select inhibitors of PD1-PDL1 interaction. For example, potential incorporation of bromine at a suitable position has been previously shown to significantly enhance the potency of PDL1 dimerizers leading to pM active compounds [12]. Finally, the adopted integrated screening protocol involving 2D fingerprint based ML models may also prove suitable for exploring other larger databases of lead- and drug-like compounds for novel PD1-PDL1 inhibitors with suitable pharmacological properties.

## Figures and Tables

**Table 1:** The ODDT training and test set modeling metrics.

Fingerprint	FP2	FP4	MACCS	Morgan	Layered	RDKit
**Training**	0.9629	0.9429	0.9549	**0.9729**	0.9649	0.9659
**Test**	0.9537	0.9283	0.9442	**0.9664**	0.9562	0.9575

**Table 2:** Top 20 virtual hits tested in the HTRF PD1-PDL1 binding assay, together with their AutoDock Vina docking scores.

Compound #	Cayman ID	% PD1-PDL1 Inhibition	AutoDock Vina Score (Kcal/mol)
1	18532	50.9 ± 1.3	−11.0
2	26113	41.4 ± 0.9	−9.6
3	19922	34.7 ± 2.1	−10.6
4	18006	24.9 ± 1.8	−9.8
5	24159	20.2 ± 1.3	−10.5
6	29424	18.7 ± 1.1	−10.3
7	19160	18.5 ± 0.7	−9.4
8	21546	17.0 ± 1.0	−9.6
9	70635	16.6 ± 1.3	−9.8
10	24057	15.9 ± 2.1	−10.6
11	32729	15.4 ± 1.3	−10.8
12	25747	14.4 ± 0.9	−10.1
13	21688	13.5 ± 1.3	−9.7
14	31758	13.1 ± 1.3	−10.4
15	21137	12.4 ± 1.5	−10.9
16	19404	5.8 ± 1.8	−9.8
17	19876	−4.3 ± 1.5	−10.4
18	18124	−4.4 ± 1.2	−10.3
19	25326	−4.5 ± 2.0	−9.9
20	17034	−4.6 ± 1.4	−10.9

## Data Availability

A publicly available database of bioactive molecules was analyzed in this study. This data can be found here: https://www.caymanchem.com/ (accessed on 2 September 2021).

## Supplementary Materials

The following are available online at https://www.mdpi.com/article/10.3390/ph15050613/s1, Table S1: List of ACTIVE, INACTIVE and DECOY molecules used for ML modeling.
